# The Potential Risk Assessment of Phenoxyethanol with a Versatile Model System

**DOI:** 10.1038/s41598-020-58170-9

**Published:** 2020-01-27

**Authors:** Mehmet Çağrı Akgündüz, Kültiğin Çavuşoğlu, Emine Yalçın

**Affiliations:** Department of Biology, Faculty of Science and Art, University of Giresun, Giresun, Turkey

**Keywords:** Abiotic, Environmental monitoring

## Abstract

In this study, the toxic effects of phenoxyethanol (Phy-Et), which is widely used in cosmetic industry, has been investigated with *Allium* test by means of physiological, cytogenetic, anatomical and biochemical parameters. To determine the changes in physiological reactions weight gain, relative injury rate, germination percentage and root length were investigated. Malondialdehyde, superoxide dismutase, glutathion and catalase levels were analyzed as biochemical parameters for determining the presence of oxidative stress. Mitotic index, micronucleus and chromosomal abnormality frequencies were studied as cytogenetic evaluation and the anatomical changes in root tip cells were investigated by cross sections. Changes in surface polarity and wettability were investigated by taking contact angle measurements of pressed root preparations. The mechanism of toxicity has been tried to be explained by these contact angles and this is the first study using contact angle measurements in toxicity tests. Consequently, exposure to Phy-Et resulted in a decrease in all measured physiological parameters and in mitotic index. In contrast, significant increases in the micronucleus and chromosomal abnormality frequencies were observed and the most significant toxic effect was found in 10 mM Phy-Et treated group. Phy-Et application induced oxidative damage and caused a significant increase in malondialdehyde level and a decrease in glutathione level compared to control group. Also a response occured against oxidative damage in superoxide dismutase and catalase activity and the activities increased in 2.5 mM and 5 mM Phy-Et treated groups and decreased in 10 mM Phy-Et treated groups. Furthermore, Phy-Et treatment resulted in some anatomical damages and changes such as necrosis, cell deformation and thickening of the cortex cell wall in root tip meristem cells of *A. cepa*. In the contact angle measurements taken against water, it was found that the wettability and hydrophilicity of the root preparations treated with Phy-Et were reduced, and this was the explanation of the growth abnormalities associated with water uptake. As a result, it was found that Phy-Et application caused toxic effects on many viability parameters and *A. cepa* test material was a reliable biomarker in determining these effects.

## Introduction

Phenoxyethanol (Phy-Et) is a aromatic glycol ether which is produced naturally in green tea and is produced by processing of phenol with ethylene oxide in the laboratory due to its commercial importance. It also known as 2-Phy-Et, phenoxetol and soluble in water, alcohol, ether and alkaline solutions. It can be miscible with propylene glycol, glycerineand benzene. Phy-Et has a molecular weight of 138.17, a melting point of 14 °C, and a boiling point of 237 °C, 242 °C, 244.9 °C and 245.2 °C. Phy-Et is widely used in the manufacture of cosmetic products such as moisturizers, hand disinfectants, soaps, sunscreen creams, mascara and perfumes because of its pure chemical form, pleasant smell and colorless appearance^1–3^. Other uses of Phy-Et include shampoos, shaving creams, ultrasound gels, insect repellents, antiseptics, solvents, anesthetics, cellulose acetate solvents, dyes, ink and ink manufacture. At higher concentrations it is also effective against microorganisms such as *Escherichia coli*, *Staphylococcus aureus* and yeasts such as *Candida*^4,5^. In addition to these benefits, Phy-Et can cause toxic effects if inhaled, ingested or skin contact in high doses. These effects include skin, lung and liver irritation, kidney and nerve damage. In addition, U.S. Food and Drug Administration reported that exposure to Phy-Et may cause dehydration, vomiting, central nervous system problems and diarrhea in infants of nursing mothers^6–8^. In a subchronic oral toxicity study performed on rats with Phy-Et, increase in body weight and feed consumption of animals and increase in liver, kidney and thyroid weights in necropsy were determined. At low concentrations Phy-Et, which has a lethal effect on many invertebrates and especially insects, can also be used as insecticide and insect repellent^9^. Many non-target organisms will be contaminated as a result of Phy-Et usage in agricultural applications. Plants are the main non-target organisms that will be affected by Phy-Et toxicity. Therefore, studies on the possible toxic effects of Phy-Et on agricultural products are needed. The aim of this study is to investigate the possible toxic effects of Phy-Et by using *in vivo Allium* test. High-build plants such as *Allium cepa* are used as bioindicators for the risk assesment of various chemicals. The use of plants as bioindicator has been standardized by the United Nations Environment Program and the Environmental Protection Agency’s (EPA) international programs. EPA and the World Health Organization acknowledge that the data from these bioassays are effective and reliable in the determination of genotoxicity^10–12^. Many cytotoxicity and genotoxicity tests have been performed with *A. cepa* and these results are consistent with other toxicity tests. Rank and Nielson^13^ reported that there was a 82% correlation between carcinogenic tests performed with rodents and *A. cepa*. Özkara *et al*.^14^ stated that *A. cepa* test is a reliable and well known test in the determination of genotoxicity and cytotoxicity and correlates well with the results obtained from eukaryotic test systems. *A. cepa* has a diploid genome (2n = 16) with a monocentric chromosome. Chromosomes are quite large and useful for detecting the karyomorphological changes. Therefore, it is particularly useful in detecting the cytotoxic, genotoxic, clastogenic and aneugenic effects of toxic substances. Genotoxic effects of chemicals are also investigated by using the main bioindicator parameters such as Mitotic index (MI), micronucleus (MN) and chromosomal abnormalities (CAs) frequencies. MI provides information on cell proliferation and refers to the ratio of the number of cells in the mitotic division to the total number of cells. In particular, MI percentage of *A. cepa* stem tip cell are severely affected by the toxicity and provide reliable results in determining the toxicity of chemical agents^15–17^. Mitotic abnormalities induced by chemicals can be tested quickly and reliably, especially in meristamic cells which have high division rates. The presence, frequency and size of the MN, the other toxicity indicator in *Allium* test, provide information about the toxicity mechanism^18,19^. The rapid germination and root elongation of meristamatic cells allows the evaluation of the lethal effects and the sub-lethal dose of compounds by using germination and growth parameters in *Allium* test^19,20^.

In the light of these data, in this study the physiological, cytogenetic, anatomical and biochemical effects of Phy-Et were investigated by using *A. cepa* test. Alterations in weight gain, germination percentage and root length were investigated as physiological parameters while malondialdehyde (MDA), glutathion (GSH), superoxide dismutase (SOD) and catalase (CAT) were analyzed as oxidative stress indicators. To determine the genotoxic effects of Phy-Et on MN frequency, MI and CAs formations were studied and anatomical damages in root tip meristem cells were investigated by croos sections. Toxicity studies should be investigated in a multi-parameter manner and all parameters tested should support each other. In this respect, the surface polarity affecting water intake of plants was also investigated to support the changes in physiological parameters. For this purpose, the contact angles of water with root layer were measured. This study will provide a different perspective for such studies and will be the first in the literature.

## Materials and Methods

### Test Material and Treatment Principles

2-Phy-Et (Product number: 77699-1 L, CAS number: 0000122996) was purchased from Sigma-Aldrich and *A. cepa* (n = 12) bulbs, approximetly 3.85 gr in weight, were supplied from a commercial market in Giresun province. Bulbs were divided into 4 groups as 1 control and 3 treatment groups.The bulbs in the control group (Group I) were germinated in tap water and the bulbs in Group II, Group III and Group IV were germinated with 2.5 mM, 5.0 mM and 10 mM doses of Phy-Et, respectively. The bulbs were soaked to related solutions in glass beakers at 24 °C for 72 hours then germinated directly in experimental conditions.

### Physiological Parameter Measurements

The physiological effects of Phy-Et were examined by germination percentage (GP), relative injury rate, root length and weight gain parameters. Data on germination were determined by analysis of germinated and non-germinated bulbs after 72 hours of application. GP and relative injury rate were calculated by using Eqs. 1 and 2^21^. The root lengths were measured on the basis of the radicle with a millimetric ruler and the weights were measured with precision scales. The weight gain was determined by calculating the differences between the weights measured before and after Phy-Et exposure^22^.

1 $${\rm{GP}}( \% )=[{\rm{Number}}\,{\rm{of}}\,{\rm{germinated}}\,{\rm{bulb}}/{\rm{Total}}\,{\rm{number}}\,{\rm{of}}\,{\rm{bulb}}]\times 100$$

2 $${\rm{Relative}}\,{\rm{injury}}\,{\rm{rate}}=[ \% {\rm{GP}}\,{\rm{in}}\,{\rm{control}}\,-\, \% {\rm{GP}}\,{\rm{in}}\,{\rm{each}}\,{\rm{group}}]/[ \% {\rm{GP}}\,{\rm{in}}\,{\rm{control}}]$$

### Cytogenetic parameter analysis

To determine the MI, MN and CAs frequecy as cytogenetic parameters, *A. cepa* root tip preparations were prepared. For this aim, root tips of the bulbs were cut 1–2 cm in length and washed with water to remove the residues. Samples were hydrolyzed with 1 N HCl for 17 minutes at 60 °C after a fixation procedure in Clarke fixative (3:ethanol/1:glacial acetic acid). The samples were transferred to a clean container and stained with acetocarmine during one night. Root tip preparations were examined for CAs, MI and MN analysis with a research microscope (IRMECO IM-450 TI) and photographed at x500 magnification^23,24^. The MN and CAs frequency was calculated by analyzing 1000 cells from each group. MI was calculated using the formula given in Equation 3and a total of 10000 cells were counted for each group.

3 $${\rm{MI}}=[{\rm{Number}}\,{\rm{of}}\,{\rm{cells}}\,{\rm{entering}}\,{\rm{to}}\,{\rm{mitosis}}]/[{\rm{Total}}\,{\rm{cell}}\,{\rm{number}}]\times 100$$

### Biochemical analysis

#### Determination of lipid peroxidation

Lipid peroxidation was measured by the method recommended by Unyayar *et al*.^25^. 0.5 g of the root tips were homogenized by adding 1 ml trichloroacetic acid (TCA) solution. The homogenates were centrifuged at 12.000 g for 24 minutes at 24 °C. In a 20% TCA solution, equal volumes of thiobarbituric acid (0.5%) and supernatant were transferred to a new tube and incubated at 96 °C for 30 minutes. Then the tubes were transferred to an ice bath and centrifuged at 10.000 g for 5 minutes.The absorbance of the supernatant was measured at 532 nm and the MDA content was expressed in μM g^−1^ FW.

### GSH analysis

For GSH analysis 0.5 g of the root tips obtained from all treatment groups were homogenized in sodium phosphate buffer. The GSH level in homogenates was measured by acid–soluble sulfhydryl level determination as described by Vecchia *et al*.^26^.

### CAT and SOD analysis

For sample extraction, 0.5 g of fresh root material was collected, washed with distilled water and homogenized in 5 mL sodium phosphate buffer (pH 7.8). The homogenates were then centrifuged at 10.500 g for 20 minutes and stored at 4 °C before the supernatant enzyme analysis.

SOD activity was analyzed by the method developed by Beauchamp and Fridovich^27^. A reaction mixture was prepared containing 1.5 mL sodium phosphate buffer (0.05 M, pH 7.8), 0.3 mL nitroblue tetrazolium chloride, 0.3 mL methionine, 0.3 mL EDTA-Na_2_, 0.3 mL riboflavin, 0.01 mL extract, 0.01 mL 4% insoluble polyvinylpyrrolidone and 0.28 mL deionized water. The reaction was initiated by placing the tubes under 215 W fluorescent lamps for 10 minutes, and reaction mixture which was not exposed to light used as control. The absorbance was recorded at 560 nm and SOD activity was expressed as U mg^−1^ FW. CAT activity was analyzed according to the procedure developed by Beers and Sizer^28^. A reaction mixture was prepared containing 0.3 mL of 0.1 M H_2_O_2_, 1.0 mL of distilled water and 1.5 mL of 200 mM sodium phosphate buffer (pH 7.8). The reaction was started by adding 0.2 mL of extract. CAT activity was measured by monitoring the reduction in absorbance at 240 nm as a result of H_2_O_2_ consumption. CAT activity was expressed as OD_240 nm_ min.g^−1^.

### Anatomical damage observations

Root tips were washed in distilled waterfor removing the residues on the surface. Then cross-sections were taken from the root tips and stained with methylene blue. Anatomical structures of each group were photographed at the x500 magnification with the research microscope^29^.

### Contact angle measurements

The water contact angle values of dry and pressed root tissue samples were determined by using a digital optical contact angle meter (Data physics OCA 15 EC) by sessile drop method at 25 °C. The size parameters of the right and left contact angles were calculated automatically from the digital image by creating a dropon the pressed root tissue surface with the help of micro syringe. Measurements were evaluated by averaging at least 6 contact angles.

### Statistical analysis

Statistical analyzes were performed using the “IBM SPSS Statistics 22 SP” package program. MDA, SOD and CAT data were shown as mean ± SE (standard error), root length, weight, MN, chromosomal damage and MI data as mean ± SD (standard deviation). The statistical significance between the means was determined by One-way ANOVA and Duncan’s test, and p value < 0.05 was considered statistically significant.

## Results and Discussion

**Figure 1:**
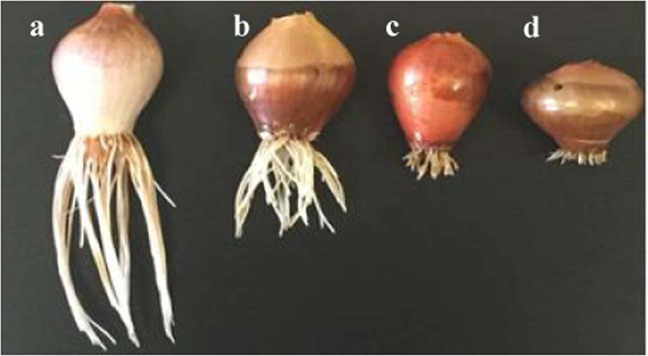
Root length views of control and Phy-Ettreatment groups (**a**): Control, (**b**): 2.5 mM Phy-Et, (**c**): 5.0 mM Phy-Et, (**d**): 10 mM Phy-Et).

Phy-Et application caused significant changes in physiological parameters and alterations observed in root length, germination percentage and weight gain are shown in Table 1. In control group germination percentage was detected as 100% and in Group IV exposed to 10 mM Phy-Et germination percentage was decreased as 50%. The highest relative injury rate rate was found in Group IV as 1.0. And also it was determined that the abnormality observed in germination percentage and injury rate was dose dependent. The changes caused by Phy-Etapplication on the root length are clearly seen in Fig. 1. It was determined that Phy-Etapplication reduced root elongation and minimum root length was observed in Group IV as 4.71 ± 1.20 cm. The root lengths of the 2.5 mM Phy-Ettreated group and 5.0 mM Phy-Ettreated group were 1.28 and 1.64 times lower than that of the control group. The decreases in root lengths were statistically significant in all three Phy-Et application groups (p < 0.05). The changes in root length and germination rates induced by Phy-Et al.so reflected to weight gain. In control group, the bulb weight increased from 3.82 ± 0.79 g to 12.15 ± 1.37 g, showing an average weight gain of 8.33 g. The weight gain of Phy-Et treated groups was behind the control group and for Group II, Group III and Group IV, the weight gain decreased by 16.6%, 38.0% and 50.7%, respectively, compared to the control group. Phy-Et is an organic chemical compound, and a kind of ether alcohol with aromatic property. Phy-Et can be broken into intermediate substrates with some enzymatic reactions such as ethylene, phenyle, ether and glycol. Phy-Et and its intermediates cause reactive oxygen species (ROS) production in some plant cells and trigger the formation of other radicals such as superoxide anion and hydrogen peroxide^30^. The toxic effects of the glycol molecules and its derivatives, another intermediate metabolite of Phy-Et, are attributed to the inhibition of phosphorus transport across the root to the xylem^31^. All these cumulative effects of Phy-Et and its intermediate molecules cause disruptions in physiological reactions, inhibition of growth and various cellular abnormalities in plants. In this study, the significant decreases in the physiological parameters observed in Phy-Et treated *A.cepa* root tip cells can be explained by the cumulative effects of these disruptions. Although there are no studies on the toxic effects of Phy-Et on plant systems in the literature, different abnormalities caused by many molecules with similar structure are reported. Plaut and Federman^32^ reported that 0.4 MPa polyethyleneglycol, a glycol derivatives, caused a 6-fold decrease in plant growth and a breakdown of chlorophyll synthesis. In a research report a decrease in body weight gain reported in subjects exposed to Phy-Et^33^. Contrary to these data, Breslin *et al*.^34^ reported that the application of ethyleneglycol phenyl ether on total body weight and tissue weights had no effect. In another study, it was reported that there were decreases in biochemical molecule levels and weight gain in Phy-Et treated subjects compared to the control group^35^.

**Table 1:** Alterations in physiological parameters of *A. cepa* induced by Phy-Et.

Groups	Germination Percentage (%)	Relative injury rate	Average root length (cm)	Final weight (g)	Weight Gain (g)
Group I	100	0.00	11.20 ± 2.28^a^	12.15 ± 1.37^a^	+8.33
Group II	83	0.17	8.75 ± 0.96^b^	10.13 ± 1.44^b^	+6.28
Group III	67	0.33	6.85 ± 1.12^c^	7.53 ± 1.07^c^	+3.72
Group IV	50	1.00	4.71 ± 1.20^d^	5.98 ± 1.48^d^	+2.12

*Group I: Control, Group II: 2.5 mM Phy-Et, Group III: 5.0 mM Phy-Et, Group IV: 10 mM Phy-Et. The averages indicated by different letters ^(a,b,c,d)^ in the same column are important at p < 0.05.

**Figure 2:**
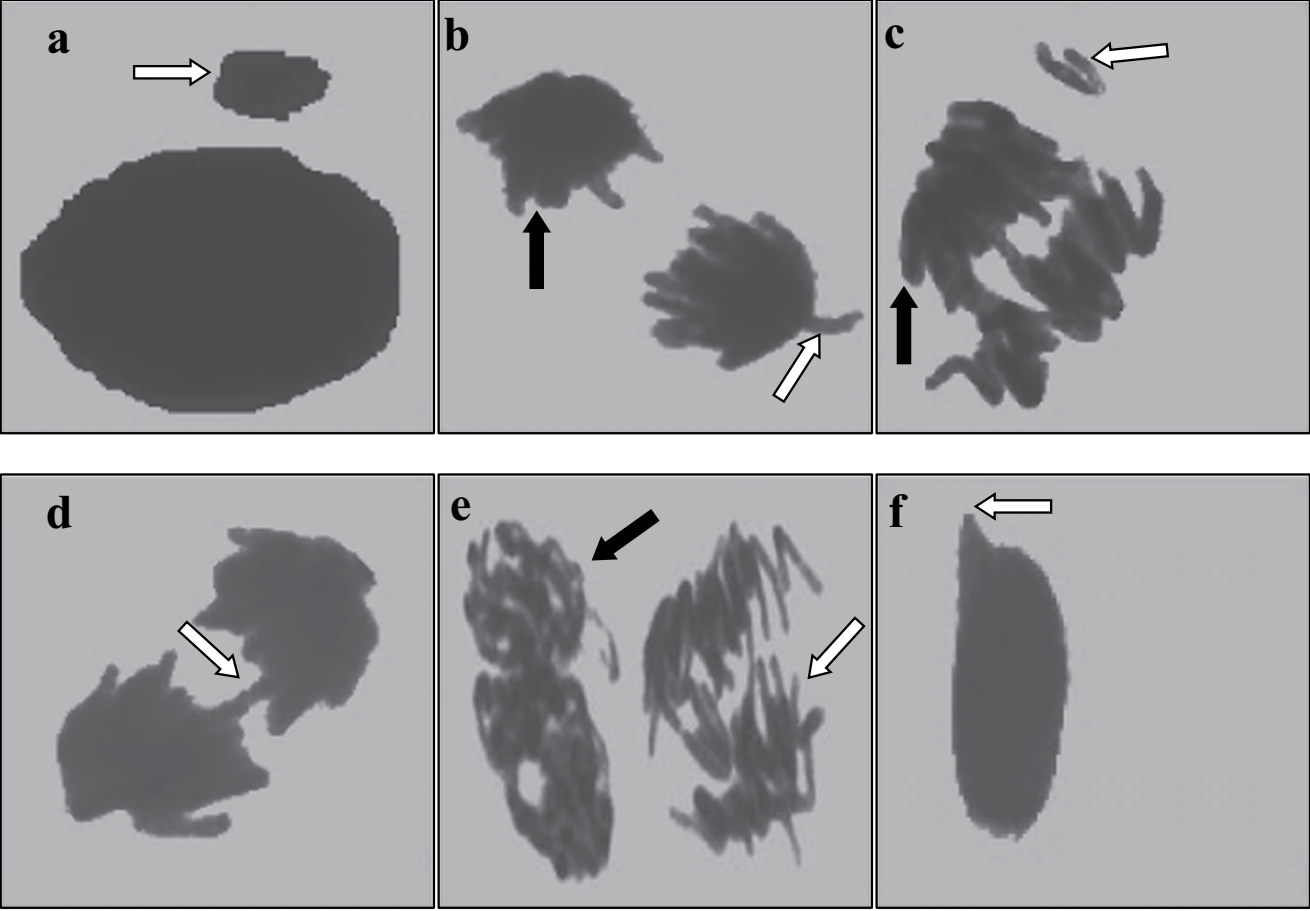
CAs formations induced by Phy-Et exposure (**a**: MN, **b**: reverse polarization [black arrow], vagrant chromosome [white arrow], **c**: fragments [white arrow], unequal distribution of chromatin [black arrow], **d**: bridge, **e**: spindle abnormality [black arrow], c-mitosis [white arrow], **f**: nucleus damage).

The effects of Phy-Et exposure on cytogenetic indicators as MI, MN and chromosomal aberrations are shown in Table 2 and Fig. 2. It was determined that MI, the indicator of cell proliferation, decreased depending on the dose of Phy-Et treatment and the most significant decrease was obtained in Group IV. 10 mM Phy-Et treatment was decreased the divided cell number from 879.20 ± 46.54 to 626.80 ± 38.88 and MI was found 1.4 times lower than control group. In control group, the frequency of MN was found as 0.20 ± 0.42 while the frequency of MN in the Group IV, which was exposed to 10 mM doses of Phy-Et, was found as 45.80 ± 3.58. The frequency of MN increased due to the increase in Phy-Et treatment dose and these increases were found to be statistically significant (p < 0.05). As a result, the observed decrease in MI rate and the increase in MN frequency indicate the cytotoxic property of Phy-Et. A decrease in MI reflects an inhibition of the cell cycle and a loss of proliferation capacity of the cell. This inhibition can be explained with many reasons such as disruption in the synthesis of macromolecules in the cell, disruption of the energy cycle, DNA damage and disruption of cell integrity. Gilbert *et al*.^4^ stated that Phy-Et application has indirect effects on ATP supplies. The formation of MN in a cell is also an indicator of the toxicity. MN is defined as formations that occur during the cell division, which do not belong to the main nucleus and originate from whole chromosome or chromosome fragments. MN is usually caused by deficiencies in genes controlling the cell cycle, errors in the mitotic spindle, kinetochore or other parts of the mitotic device and chromosomal damage^36,37^. MNs resulting from a toxic effect appear after completion of karyokinesis while chromosomal abnormalities can be observed at any stage of the cell cycle. In this study, after Phy-Et exposure different kinds of chromosomal aberrations were observed such as c-mitosis, chromosome bridge, vagrant chromosome, fragment, reverse polarization, spindle abnormality and unequal distribution of chromatin (Fig. 2). Only a few vagrant chromosome, c-mitosis and spindle abnormality were observed in root tip cells of the control group without statistical significance. The highest CAs frequency was observed in 10 mM Phy-Et treatment and it was determined that all CAs formations increased with Phy-Et treatment dose increased. Fragment and vagrant chromosome were the most common CAs observed (Fig. 2b,c) in this study. The fragment is formed by a breakage and rupture of the phosphodiester backbone of DNA. The consequences of the breakage are severe and this can lead to new rearrangements such as MN formations, translocations, inversions and deletions. Although there are no studies investigating the effects of Phy-Et on plant chromosomes, some studies performed with AMES test did not report a mutagenic effect of Phy-Et at 5000 μg/plate dose. Similarly, Phy-Et has been reported to have no clastogenic effect in the *in vitro* structural chromosome aberration test^38^. In contrast to studies indicating that Phy-Et is not mutagenic and clastonegic, there are also studies supporting our results. Gilbert *et al*.^4^, proposed that 2-Phy-Et may have a direct inhibitory effect on macromolecule biosynthesis, DNA and RNA synthesis and an indirect effect on adenine triphosphate (ATP) supplies. Serious alterations in DNA synthesis and ATP production in a cell will also affect mitotic activity. In literature genotoxic effects of chemicals similar to Phy-Et structure are mentioned. Phenyl ether, a Phy-Et derivative, has been reported to cause mitotic recombination, gene conversion, and gene reversion without metabolic activation^39^.

**Table 2:** Alterations in cytogenetic parameters induced by Phy-Et.

Cytogenetic parameter	Group I	Group II	Group III	Group IV
MI (%)	8.79	8.30	7.48	6.26
Divided cell Number	879.20 ± 46.54^a^	830.10 ± 27.58^b^	748.00 ± 61.47^c^	626.80 ± 38.88^d^
MN Frequency	0.20 ± 0.42^d^	7.00 ± 1.83^c^	16.60 ± 2.67^b^	45.80 ± 3.58^a^
Fragment	0.00 ± 0.00^d^	10.10 ± 2.88^c^	24.60 ± 4.62^b^	52.70 ± 4.19^a^
Vagrant chromosome	0.20 ± 0.42^d^	8.80 ± 2.04^c^	17.10 ± 3.18^b^	38.20 ± 3.74^a^
Chromosome bridge	0.00 ± 0.00^d^	6.80 ± 1.81^c^	12.00 ± 3.40^b^	31.40 ± 4.35^a^
C-mitosis	0.30 ± 0.48^d^	3.50 ± 1.58^c^	9.30 ± 3.50^b^	16.90 ± 3.14^a^
Nucleus damage	0.00 ± 0.00^d^	3.00 ± 1.76^c^	6.80 ± 1.87^b^	12.00 ± 2.83^a^
Reverse polarization	0.00 ± 0.00^d^	2.90 ± 1.37^c^	5.10 ± 1.52^b^	10.40 ± 2.72^a^
Spindle abnormality	0.20 ± 0.42^d^	2.10 ± 1.10^c^	4.10 ± 1.66^b^	8.30 ± 2.45^a^
Unequal distribution of chromatin	0.00 ± 0.00^d^	5.70 ± 2.06^c^	11.00 ± 2.36^b^	24.20 ± 3.39^a^

*Group I: Control, Group II: 2.5 mM Phy-Et, Group III: 5.0 mM Phy-Et, Group IV: 10 mM Phy-Et. Data were shown as mean ± SD (standard deviation) (n = 10). Data were analyzed with SPSS computer program using Duncan test and ANOVA variance analysis. The averages indicated by different letters ^(a,b,c,d)^ in the same line are important at p < 0.05.

**Figure 3:**
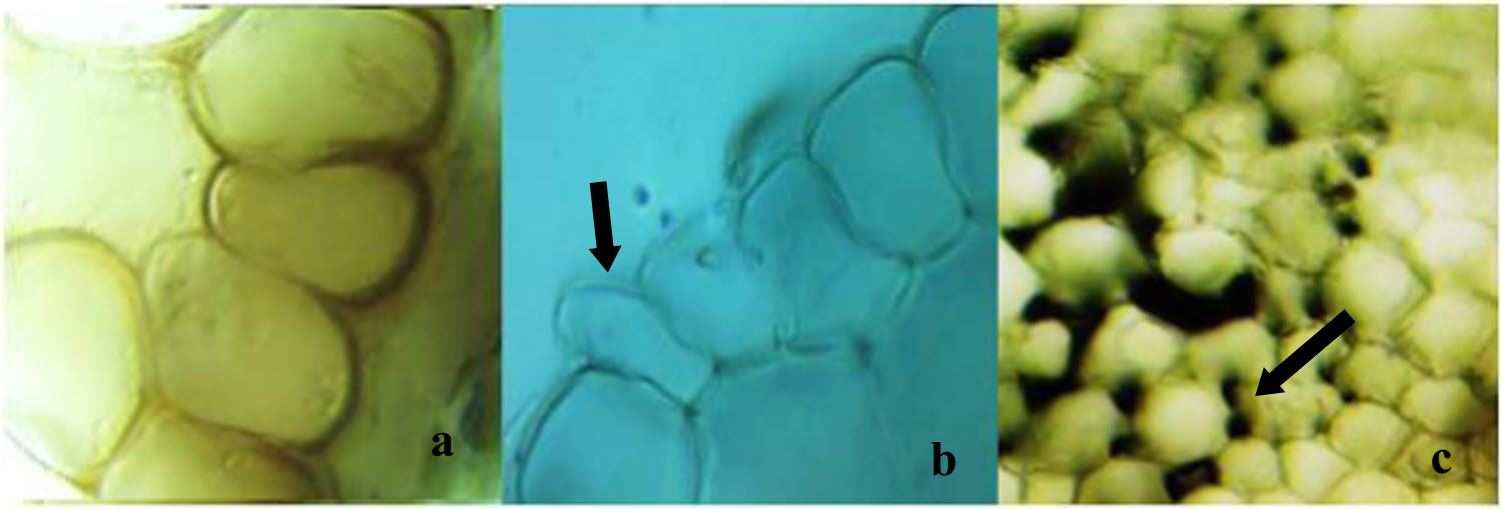
Anatomical damages caused by Phy-Et exposure (**a**: necrosis, **b**: epithelial cell deformation, thickening of the cortex cell wall).

The anatomical changes and damages induced by Phy-Et in the root tip meristematic cells are shown in Fig. 3. In microscopic examinations, no anatomical damage was observed in the meristematic cells of the root tip of the control group. Anatomical damages and changes such as necrosis, epithelial cell deformation and thickening of cortex cell wall were observed in root tips of the groups exposed to Phy-Et. The thickening of cortex cell observed as anatomical changes can be related to the adaptation mechanism developed by the tissues and cells against the Phy-Et treatment. In order to tolerate the toxic effects of chemicals, plants develop mechanisms such as reduced substance transport, thickening of cortex cells and accumulation of matter in the cell wall^40^. As a result of these mechanisms, anatomical changes occur in the plant and the toxic effects of chemical agents are reduced. For example, Liu *et al*.^41^, reported that the cell wall thickening occurs as a result of toxicity in *Vicia faba* and this anatomical change restricts the transport of toxic substances to other cells. However, in some cases the high toxic effect causes anatomical damage rather than anatomical change. The fact that necrosis and cell deformations observed in this study was dominant in the 10 mM Phy-Et treatment group confirms this hypothesis. In literature, no data has been reported on Phy-Et effect on plant anatomy.

**Figure 4:**
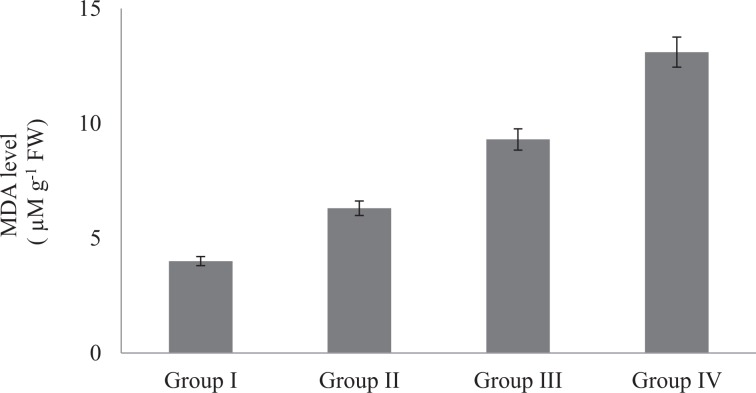
Root MDA levels of control and Phy-Et administration groups(Group I: Control, Group II: 2.5 mM Phy-Et, Group III: 5.0 mM Phy-Et, Group IV: 10 mM Phy-Et).

**Figure 5:**
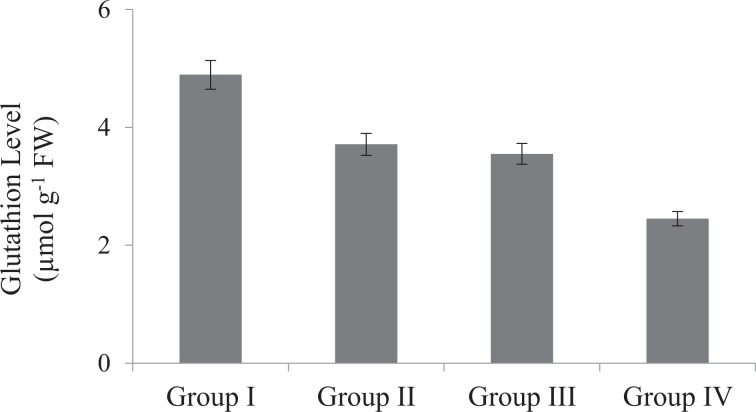
Root GSH activities of control and Phy-Et administration groups (Group I: Control, Group II: 2.5 mM Phy-Et, Group III: 5.0 mM Phy-Et, Group IV: 10 mM Phy-Et).

**Figure 6:**
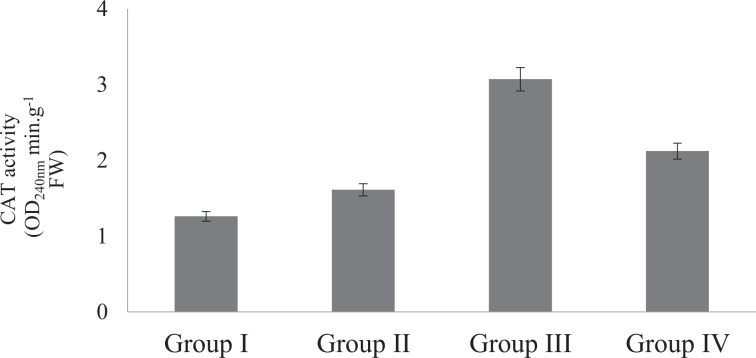
Root CAT activities of control and Phy-Et administration groups (Group I: Control, Group II: 2.5 mM Phy-Et, Group III:5.0 mM Phy-Et, Group IV: 10 mM Phy-Et).

**Figure 7:**
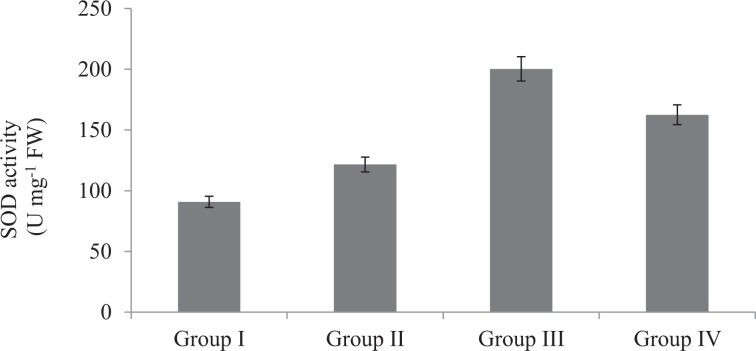
Root SOD activities of control and Phy-Et administration groups (Group I: Control, Group II: 2.5 mM Phy-Et, Group III: 5.0 mM Phy-Et, Group IV: 10 mM Phy-Et).

The effects of Phy-Et treatment on root MDA levels, which is an important indicator of lipid peroxidation, are shown in Fig. 4. While the presence of MDA in the control group was found to be 4.0 µmolg^−1^, the dose-related increase in MDA level was observed after Phy-Et application. Maximum MDA level was observed in 10 mM Phy-Et treated group as 13.10 µmolg^−1^. When compared with the control group, MDA levels in Group II, III and IV were increased 1.58 times, 2.33 times and 3.28 times, respectively and these increases were statistically significant (p < 0.05). Phy-Et application caused an increase in MDA levels as well as a decrease in GSH level (Fig. 5). It was determined that the level of GSH decreased with the application of Phy-Et and the most significant decrease was observed in Group IV. In 10 mM Phy-Et treated group, the mean GSH level decreased 49.9% compared to control group. The effects of Phy-Et exposure on CAT and SOD activity of *A. cepa* stem cells are shown in Fig. 6 and Fig. 7. When antioxidant enzyme activities were examined, it was determined that Phy-Et application caused a dose dependent change in SOD and CAT activities.CAT activity was found 1.26 OD_240nm_min g^−1^ in control group and increased 2.43 times in 5.0 mM Phy-Et treated group compared to the control group. However, CAT activity decreased in 10 mM Phy-Et treated group and regressed to 2.12 OD_240nm_min g^−1^. A similar effect was observed in the SOD activity and the highest SOD activity was measured to be 200.3 U mg^−1^ FW in 5.0 mM Phy-Et treated group and it was found to decrease to 162.5 U mg^−1^ FW after 10 mM Phy-Et treatment. In summary, it was determined that CAT and SOD enzyme activities increased due to the oxidative stress formation induced by 2.5 mM and 5.0 mM Phy-Et treatments. However, due to the enzymatic structure deformation caused by the 10 mM Phy-Et treatment, the enzyme activities decreased. Phy-Et administration caused changes in antioxidant homeostasis of *A. cepa* root cells. The increase in MDA level, decrease in GSH level and the fluctuations in antioxidant enzyme activities are associated with Phy-Et toxicity. Phy-Et and its intermediate molecules cause ROS, superoxide anion and hydrogen peroxide production plants cells^30^. MDA is a three-carbon low molecular weight aldehyde and produced after the breakdown of polyunsaturated fatty acids by the free radical attack^42^. An increase in the level of MDA indicates the presence of oxidative damage in cell and in this case antioxidant enzymatic activities are induced. The increase in the MDA level after Phy-Et application is an evident of oxidative damage. Enzymes and non-enzymatic antioxidant molecules are involved to neutralize this damage. GSH is a non-enzymatic tripeptide antioxidant and an important part of antioxidant defense in the removal of free radicals. Reduced GSH in the cell neutralizes free radicals and protects the cell from oxidative damage. As a result of this reaction, GSH molecules are converted to oxidized GSSG state and the amount of GSH decreases^43^. Briefly, an increase in the MDA rate and a decrease in the GSH rate prove the oxidative damage induced by Phy-Et. In addition to non-enzymatic antioxidants, there are also defense strategies against radical attack and oxidation in cells including enzymatic antioxidants such as SOD and CAT. Antioxidant enzymes are the primary mechanism for protecting cells against oxidative damage *in vivo*. SOD is induced in the presence of superoxide anion and converts the free radical into H_2_O_2_ and O_2_ in order to eliminate the radicalic effect. H_2_O_2_ formed as a result of the SOD reaction is decomposed into H_2_O and O_2_ by CAT activity and thus, the oxidative effect is eliminated^44^. In contrast to the increase in 2.5 and 5.0 mM Phy-Et applications, enzymatic activities decreased in 10 mM Phy-Et application. It is thought that this decrease is associated with the high doses of Phy-Et causing denaturation of protein structures of SOD and CAT enzymes. Although the effects of Phy-Et on antioxidant system in plants have not been studied in the literature but some studies have reported that there are changes in various biochemical parameters of organisms exposed Phy-Et. Priborsky *et al*.^45^ reported that Phy-Et reduces SOD activity and increases CAT activity in *Barbus barbus*. Uçar *et al*.^46^ reported that there were significant changes in the activity of glutathione reductase and catalase in *Oncorhynchus mykiss* and *Salmo trutta fario* species exposed to Phy-Et. In a study, Phy-Et has been reported to cause irreversible oxidation of macromolecules in *P.auregonisa*^47^. Velíšek *et al*.^48^ reported that Phy-Et treatment induces the formation of reactive oxygen species, leads to oxidative damage to macromolecules and causes a decrease in antioxidant capacity.

**Figure 8:**
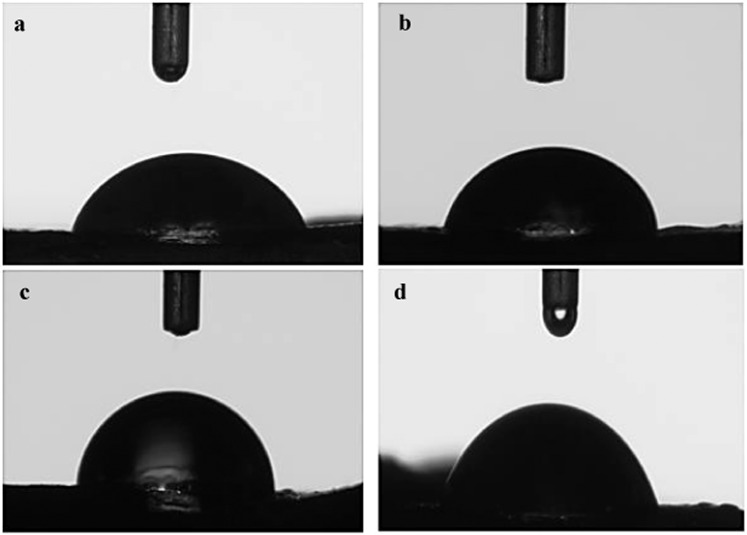
Contact angle measurements of root samples (**a**) Control, (**b**) 2.5 mM Phy-Et treated group, (**c**) 5 mM Phy-Et treated group, (**d**) 10 mM Phy-Et treated group.

The contact angles of the root tissues against water are given in Fig. 8. Contact angle measurements are used to evaluate surface polarity and wettability. Phy-Et treatment caused important changes in polarity and more importantly wettability of the root tissues. In the control group the contact angle of was found as 67.9°, whereas in the 10 m MPhy-Et treated group the contact angle was increased 1.25 times and found as 85.5°. The increase in contact angle with water may be associated with a change in surface polarity and consequently a decrease in hydrophilicity^32^. The decrease in hydrophilicity can be explained mainly by the difference in chemical properties and morphology of the cell surface. The first interaction of chemicals with root tissue takes place on the merictamic cell surface and damage begins here. Phy-Et has a particularly radical effect, causing damage to the cell membrane, lipid peroxidation and deteriorating membrane integrity. Lipid peroxidation may lead to changes in cell membrane structure and thus change the hydrophilic character of cell surface.Water uptake is essential for root development and plant growth, and roots play an important role in water uptake. A change in the structure of the root will prevent water uptake and the transfer of the water into the cell so abnormalities in plant growth and germination occur. The decrease in water transfer to cells will also decrease proliferation, thus affecting MI rates. The results of the contact angle measurements and the physiological abnormalities observed in the Phy-Et treated groups and the changes in MI support each other.

## Conclusion

With the increase in technology and industrialization, exposure of all living things to toxic agents has increased intensively. Therefore, studies investigating the toxic effects of each agent have gained considerable importance. Phenoxyethanol, which is included in personal care products and cosmetics, is a frequently exposed agent and its effects on living organisms are not fully elucidated. In this study, the toxic effects of phenol in *A. cepa*, which is a eukaryotic model organism, were investigated with a multi-parameter approach. As a result, phenol was found to cause serious damage especially at 10 mM dose in terms of cytogenetic, physiological, biochemical and anatomical parameters. Therefore, limiting the use of Phy-Et, if possible not preferred or in cases where it is absolutely necessary, appropriate dose levels should be determined which will not have a toxic effect on humans.In the light of these data, it should be ensured that the use of Phy-Et should be limited, if not preferred, or if it is absolutely necessary to use it in doses that do not have toxic effects on the organisms.
